# Supplementary material for: Sex Differences in Complex Posttraumatic Stress Disorder Network among Chinese Young Adults
Source: Behav Sci (Basel). 2023 Oct 16;13(10):846. doi: 10.3390/bs13100846 (PMC10604758; doi:10.3390/bs13100846)

Figure S1. Bootstrapped confidence intervals (CIs) of the edge weights of the CPTSD symptom networks of men (a) and women (b). The red line indicates the edge weight values, and the gray area indicates the 95% CIs.

(a)

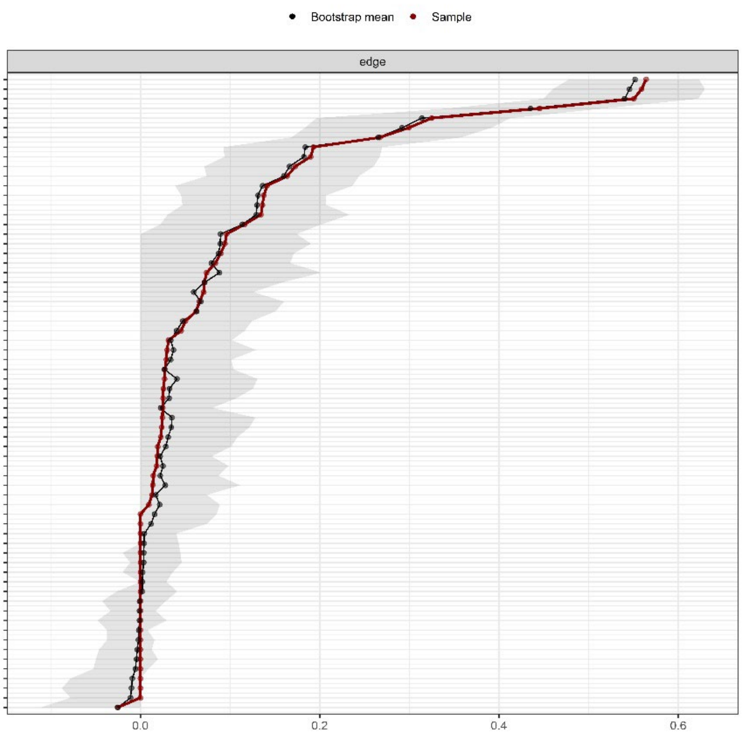

(b)

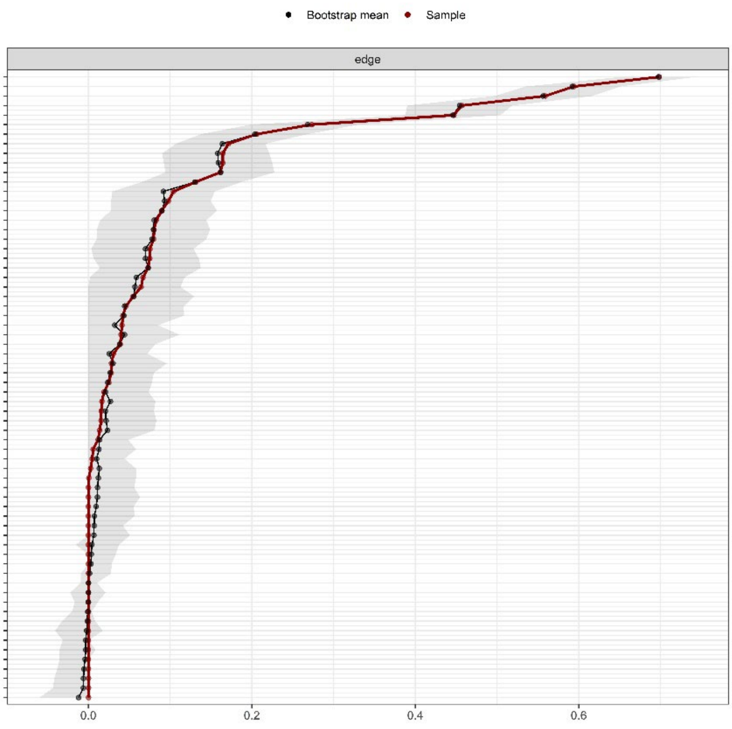

Figure S2. Subsetting bootstrap for the CPTSD networks of men (a) and women (b). Figures show the average correlations between centrality indices of the original sample and subsamples with fewer participants.

(a)

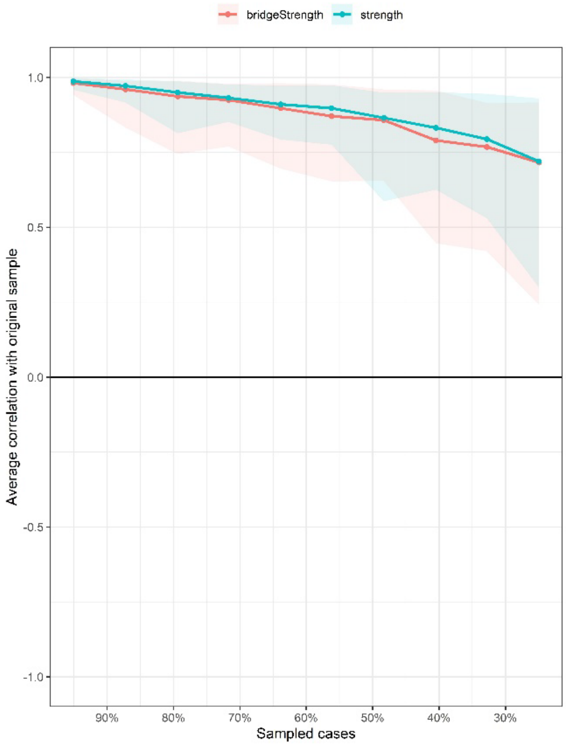

(b)

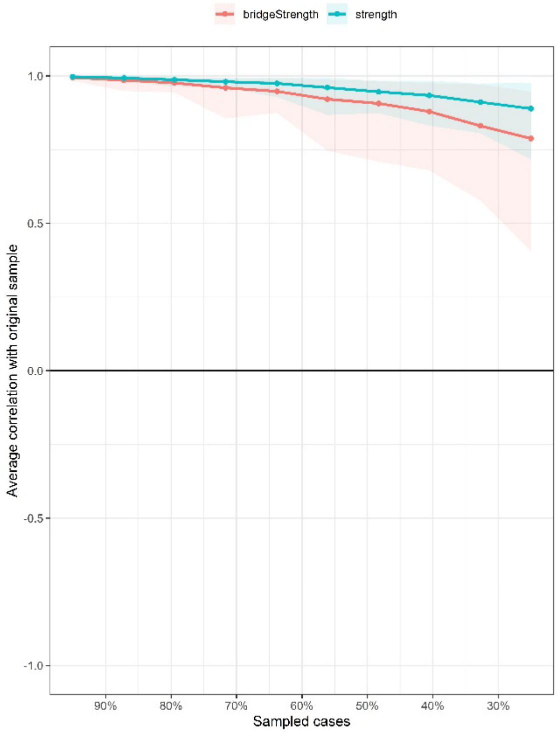

Figure S3. Edge weight difference tests for the CPTSD symptom networks of men (a) and women (b). The black boxes represent an edge that differs significantly from another one (α = 0.05).

Notes: RE1: nightmares; RE2: flashbacks; AV1: internal avoidance; AV2: external avoidance; TH1: hypervigilance; TH2: exaggerated startle response; AD1: long-time upset; AD2: emotional numbing; NSC1: feelings of failure; NSC2: feelings of worthlessness; DR1: feeling distant or cut off from others; DR2: difficulties feeling close to others.

(a)

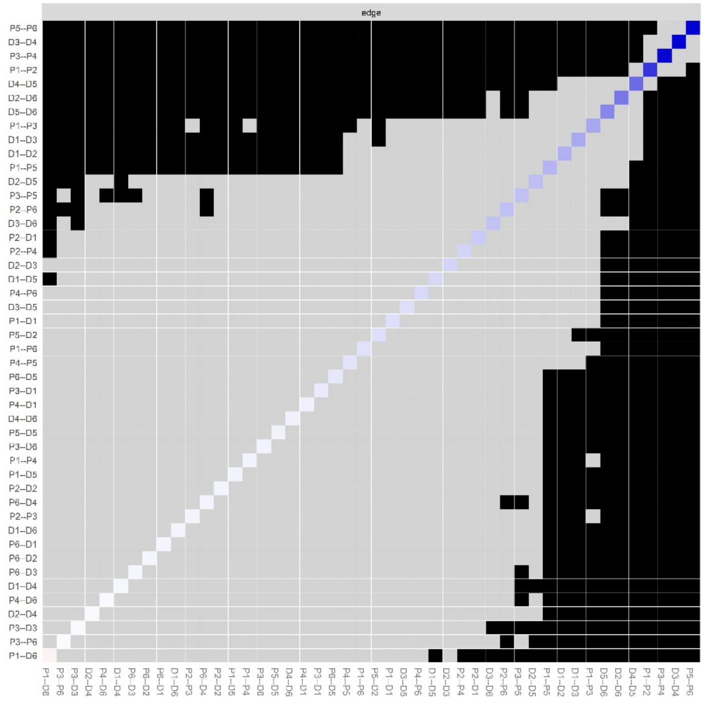

(b)

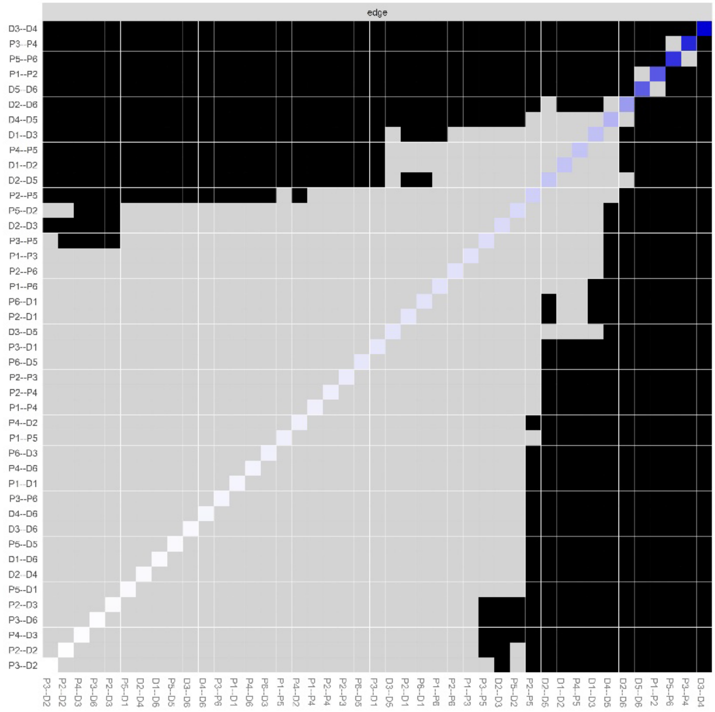

Figure S4. Node expected influence difference tests for the CPTSD symptom networks of men (a) and women (b). The black boxes represent a node that differs significantly from another node (α = 0.05).

Notes: RE1: nightmares; RE2: flashbacks; AV1: internal avoidance; AV2: external avoidance; TH1: hypervigilance; TH2: exaggerated startle response; AD1: long-time upset; AD2: emotional numbing; NSC1: feelings of failure; NSC2: feelings of worthlessness; DR1: feeling distant or cut off from others; DR2: difficulties feeling close to others.

(a)

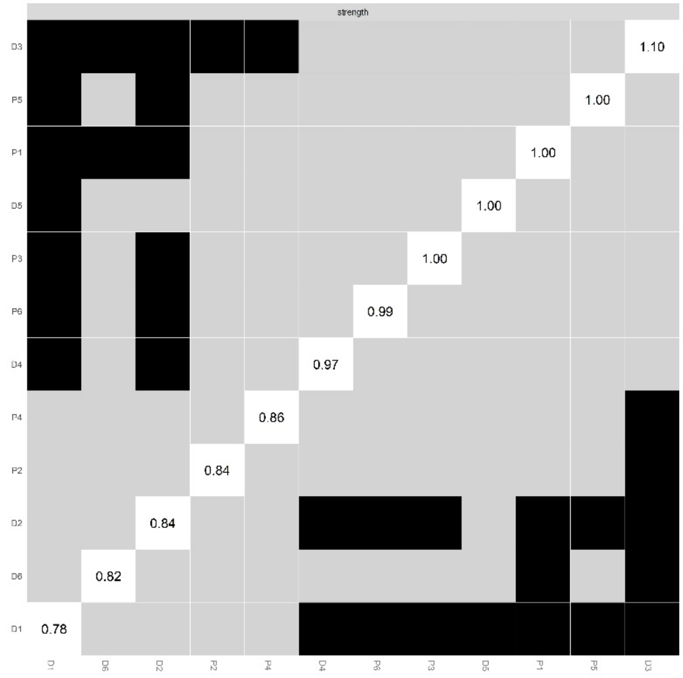

(b)

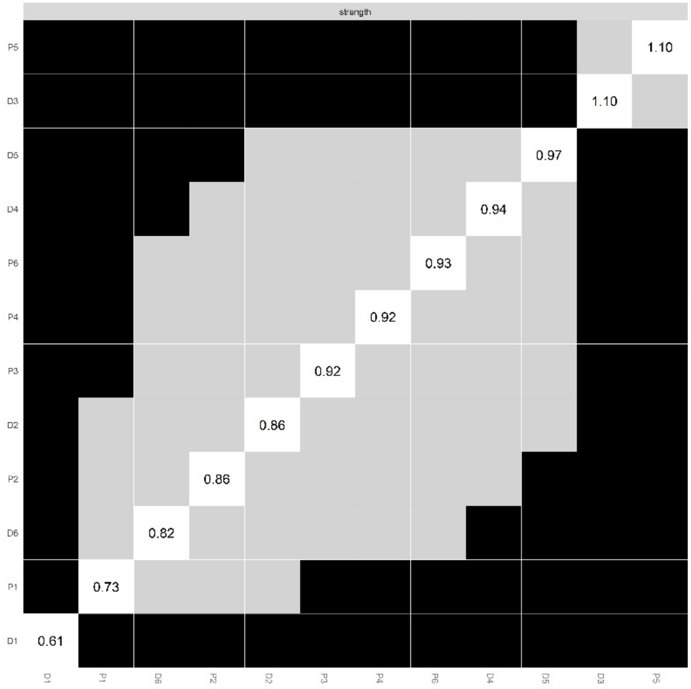

Supplement: Supplementary file 1 [file behavsci-13-00846-s001.zip › behavsci-2613111-supplementary.pdf]
